# Study on the evaluation method of pilot workload in eVTOL aircraft operation

**DOI:** 10.1016/j.heliyon.2024.e37970

**Published:** 2024-09-16

**Authors:** Lijuan Hu, Xufei Yan, Ye Yuan

**Affiliations:** a Zhejiang Financial College, Hangzhou, 310018, China; b Tianmushan Laboratory, Hangzhou, Zhejiang Province, 311100, China; c Swansea University, Swansea, Wales, SA2 8PP, UK

**Keywords:** eVTOL aircraft, Pilot control workload, Wavelet transform, Frequency component, HQR

## Abstract

This paper investigates the pilot control strategy and workload associated with the manned electric Vertical Take-Off and Landing (eVTOL) aircraft. This research aims to identify control strategies that are both rational and effective in reducing the pilot's workload. Although the Cooper-Harper rating (HQR, Handling Quality Rating) remains the prevalent benchmark for assessing pilot workload, its subjective nature often complicates the precise quantitative identification of the key factors influencing workload. This study introduces an assessment method for pilot control workload using wavelet transform to evaluate pilot workload, focusing on the correlation between pilot control magnitude, frequency components, and complexity. We begin with a review of control action analysis methods in both time and frequency domains. Subsequently, by selecting an appropriate wavelet function and a sampling frequency, we develop the assessment method for pilot control strategy and workload based on wavelet analysis. Finally, we assess pilot workload using real pilot inputs with HQRs obtained from helicopter flight tests and tilt-rotor aircraft conversion simulations. The results indicate that this approach is capable of precisely pinpointing the frequency components and energy levels within pilot control actions throughout different time periods. Furthermore, a significant correlation is observed between pilot control characteristics, frequency components, and HQRs. Consequently, the developed approach provides a rational framework for quantifying and analyzing pilot workload in a range of eVTOL aircraft scenarios.

**Table:** Nomenclature

$G_{\delta \delta }$	power spectral density (PSD)
$n_{c}$	the number of cycles
s	scaling factor
t_0_	initial time, s
u	translation parameter
$W_{x}$	aggressiveness of the pilot manipulation of a specific control channel
$W_{\text{tot}}$	sum of the aggressiveness values for all control inputs
$W_{y}\left(u,s\right)$	wavelet coefficient
y(t)	time-varying signal
$\delta_{x}\left(t\right)$	time history of the pilot control input for a particular channel
$\delta_{col}\left(t\right)$, $\delta_{lon}\left(t\right)$	collective stick input, and longitudinal cyclic stick input
$\delta_{x,\text{trim}}$	trim control stick position
$\delta_{\text{xmax}}$	maximum control input
$\delta_{\text{xmin}}$	minimum control input
ω	frequency
$\omega_{co}$	cut-off frequency
$\sigma_{\text{total}}$	root mean square (RMS) within the entire frequency domain range
ψ(t)	wavelet function

## 1 Introduction

Over the past few years, the concept of Urban Air Mobility (UAM) has attracted significant interest due to its promise for operations in low-altitude flight, transportation, and specialized aerial missions [1,2]. Aircraft with electric vertical take-off and landing (eVTOL) technology have become the focus within this domain. Their ability to take off and land vertically, along with enhanced safety features and reduced noise emissions, makes them a promising solution for achieving sustainable urban transportation. While eVTOL aircraft have entered the autonomous control phase, it is important to note that manual control remains an essential aspect according to aviation regulations worldwide. Currently, the mainstream control method acknowledged by civil aviation authorities involves a combination of manual pilot control and automatic control stability system. Hence, studying the pilot control strategy and workload for manned eVTOL aircraft is extremely important. This research aims to identify control strategies that are both rational and effective in reducing the pilot workload.

Pilot control workload, which refers to the cost incurred by the pilot to accomplish specific flight tasks, is a crucial factor in assessing flight quality and the rationality of the control strategy. Currently, the pilot control workload assessment primarily relies on the pilot-in-loop simulations or flight experiments, with Cooper-Harper's subjective rating (HQR, Handling Quality Rating) remaining the main standard for such analysis. However, the HQR often struggles to determine the primary factors and their impact on control workload accurately and quantitatively. Therefore, in recent years, many researchers have attempted to define methods for quantifying the pilot control workload [3]. Although the flight quality specification ADS-33E provides flight quality ratings for specific helicopter maneuvers, there is currently no standardized evaluation criterion for assessing pilot control workload in eVTOL aircraft [2].

Previously, researchers primarily employed basic metrics for analyzing pilot control actions, such as average control input magnitude [4], aggressiveness, average duration of stable control, and frequency of control actions [5,6]. Advanced techniques, such as the pilot's classical cut-off frequency, Power Spectral Density (PSD), and root mean square (RMS), have been utilized to characterize pilot control workload, simulator fidelity, and the correlation between pilot input frequency and task demands [7,8]. However, these metrics have demonstrated limited accuracy in assessing pilot control workload when compared to flight tests involving actual pilots. To address this limitation, Gray [9] proposed a two-dimensional representation known as the "Pilot Workload Image" as an alternative to existing pilot control action analysis methods, with "control cycle" and "magnitude of rapidness" labeled on the *x* and *y* axes, respectively. While Gray did not provide a specific mathematical expression, he observed that the pilot control workload escalates as the computed points diverge from the origin. Additionally, this method of representation could aid in comparing the control workload among various pilots and their respective attempts to perform the same task. Gray's approach is considered valid because it acknowledges that the workload is primarily influenced by the magnitude and frequency of the pilot's control inputs. However, a limitation of traditional methods for analyzing pilot control actions is their restricted ability to consider the temporal variations inherent in these actions.

With the increasing computational capabilities and advancements in signal processing theory, recent research on quantifying pilot control workload has focused on methods that characterize the temporal characteristics of control actions. Among the various methodologies, wavelet transform-based time-frequency representation (TFR) and the short-time Fourier transform (STFT) have been extensively developed within the field of signal processing and are routinely utilized for analyzing pilot control actions. Jones and Padfield [10] utilized wavelet analysis to extract frequency and amplitude information from flight test data. They demonstrated that this information can serve as suitable indicators for the "aggressiveness" and "agility" parameters in low-level helicopter flight tasks. Klyde et al. [3,11,12] employed wavelet analysis to detect oscillations caused by pilots in fixed-wing aircraft and introduced a technique for measuring the workload of pilot control. This method utilizes time-frequency scale maps obtained through wavelet analysis to derive power frequency parameters. Tritschler and O'Connor [13] introduced a technique for multi-frequency component analysis based on the short-time Fourier transform, which facilitates the detection of the most significant frequency components within pilot control inputs associated with their control strategies. They pointed out the potential correlation between the frequencies of pilot control actions and flight tasks. In response to this, Tritschler et al. [14] have utilized a range of methods for analyzing pilot control actions to conduct a detailed examination of pilot behaviors in the context of hover and autorotation flight missions. The results indicated that the time-varying cut-off frequencies may not accurately capture the details of the actual control input frequencies used by pilots in certain flight tasks. Conversely, the primary frequency components extracted via multi-frequency component analysis closely match pilots' descriptions and provide supplementary insights into control frequencies and energy distribution.

In summary, the current theoretical methods for evaluating pilot control strategy and workload can be categorized into three main groups: time-domain analysis, frequency-domain analysis, and time-frequency representation methods [15].

(1) Time-Domain Evaluation Methods

The time-domain evaluation methods, including average control input magnitude, aggressiveness, and average duration of stable control, analyze pilot control inputs over a specific period to capture the magnitude and timing of their actions. However, these methods pose challenges in accurately reflecting the frequency of pilot control.

(2) Frequency-Domain Evaluation Methods

The frequency-domain evaluation methods encompass pilot classical cut-off frequency, Power Spectral Density (PSD), and root mean square (RMS). These methods are focused on examining the frequency components present in pilot control inputs. By employing techniques such as Fourier transform or wavelet transform, we can identify the dominant frequencies and their energy levels. However, a limitation of these approaches is their restricted capacity to account for temporal fluctuations within these control actions.

(3) Time-Frequency Representation Evaluation Methods

The time-frequency representation evaluation methods encompass the pilot workload image, the power frequency that varies over time, and the analysis of multiple frequency components. Although the pilot workload image lacks a precise mathematical formulation, it is chiefly affected by the magnitude and frequency of the pilot's operational inputs. The time-varying power frequency and the multi-frequency component rely on short-time Fourier transform or wavelet transform, which enable a thorough visualization of signal energy distribution across both frequency and time domains. However, the time-varying power frequency is unable to accurately identify specific pilot operational frequency components, making it difficult to accurately evaluate pilot workload. In contrast, the multi-frequency component can identify pilot operational frequency components and provide a more accurate quantification and evaluation of pilot control workload. This method has not been further investigated to quantify pilot control workload using simple indicators. Therefore, to date, no comprehensive indicator has been established to evaluate pilot control workload.

Drawing on established pilot control action analysis methods, this paper aims to present an evaluation approach for pilot control workload. It utilizes wavelet transform to examine the relationship between control magnitude, frequency components, and associated control difficulty. This approach offers a thorough portrayal of pilot control inputs with respect to amplitude and frequency, and further translates the pilot workload into a straightforward indicator, the HQR, thereby merging both qualitative and quantitative analytical viewpoints. Since this technique is grounded in the evolution of control action analysis methods in both the time and frequency domains, we initially offer an overview of these two methodologies. Then, we focus on modeling the evaluation method for pilot control workload based on wavelet transform. Finally, we evaluate the pilot control workload using actual pilot control inputs and HQRs [16] in a flight test of a helicopter (UH-60A) single-engine failure safe landing, and a tilt-rotor aircraft conversion simulation [15] to demonstrate the validity of this method, addressing the absence of publicly available pilot control data and corresponding workload evaluations for eVTOL aircraft.

## 2 Time-domain and frequency-domain evaluation methods

Within this section, we deliver a concise summary of the existing theoretical methodologies (time-domain and frequency-domain methods) for evaluating pilot performance, offering valuable insights into pilot control strategies and workload. Subsequently, to demonstrate the limitations of current methods for assessing pilot control workload, we use these indicators to calculate three types of maneuvers performed by the UH-60A helicopter and compare them with the actual HQRs provided by the pilots.

### 2.1 Aggressiveness

In the time-domain evaluation, we analyze the pilot control inputs over a specific period to capture the magnitude and timing of their actions. This analysis helps us understand how pilots execute maneuvers and respond to different flight conditions. Aggressiveness is a time-domain method that quantifies the level of dynamic control manipulation and the deviation from trim position during maneuvering. It is defined as the integration of the deviation quantity over time, followed by time averaging and normalization. Mathematically, aggressiveness can be expressed as follows [5],

(1) $$\begin{array}{l} W_{x}=\frac{100\%}{t_{f}-t_{0}}\int_{t_{0}}^{t_{f}}\left|\frac{\delta_{x}\left(t\right)-\delta_{x,\text{trim}}\left(t_{0}\right)}{\delta_{\text{xmax}}-\delta_{\text{xmin}}}\right|dt\\ W_{\text{tot}}=\sum W_{x} \end{array}$$

In Equation (1), $W_{x}$ represents the aggressiveness of the pilot's manipulation of a specific control channel; $\delta_{x}\left(t\right)$ denotes the temporal record of the pilot's control input for a specific channel; $\delta_{x,\text{trim}}\left(t_{0}\right)$ represents the trim control stick position at time *t*_0_. It indicates the neutral or desired position of the control stick for maintaining steady flight or equilibrium. This value serves as a reference point for evaluating the deviation of control inputs from the trim position during maneuvering; $\left|\delta_{\text{xmax}}-\delta_{\text{xmin}}\right|$ is the maximum displacement value of the control input; $W_{\text{tot}}$ is the sum of the aggressiveness values for all control inputs, which measures the overall magnitude variation of the pilot's control inputs during maneuvering.

### 2.2 Pilot cut-off frequency

On the other hand, frequency-domain evaluation focuses on examining the frequency components present in pilot control inputs. By using techniques such as Fourier transform or wavelet transform, we can identify the dominant frequencies and their energy levels. This allows us to gain insights into the control strategies employed by pilots and the distribution of control efforts across different frequency bands.

The pilot cut-off frequency is a frequency-domain method. The greater the difficulty of a pilot's maneuver, the higher the pilot's cut-off frequency. Moreover, the pilot cut-off frequency can offer a reliable estimate of the pilot's cross-frequency during envelope tasks. Spectral analysis methods can be employed to scrutinize the temporal sequence of pilot control inputs, thereby ascertaining the operational or cut-off frequency pertinent to the pilot. Assuming a control input time history $\delta_{x}\left(t\right)$, its power spectral density (PSD) denoted by $G_{\delta \delta }\left(\omega \right)$ in the frequency range of $0∼\omega_{1}$, and the root mean square (RMS) of pilot control input signal within this range represented by $\sigma_{1}$, the following formula [16] can be obtained as shown in Equation (2),

(2) $$\sigma_{1}^{2}=\frac{1}{2\pi }\int_{0}^{\omega_{1}}G_{\delta \delta }\left(\omega \right)d\omega$$

The expression for the root mean square (RMS) within the entire frequency domain range can be given by Equation (3),

(3) $$\sigma_{\text{total}}^{2}=\frac{1}{2\pi }\int_{0}^{\infty }G_{\delta \delta }\left(\omega \right)d\omega$$

The pilot's cut-off frequency $\omega_{co}$ can be defined as shown in Equation (4),

(4) $$\frac{\sigma_{\text{co}}^{2}}{\sigma_{\text{total}}^{2}}=\frac{\frac{1}{2\pi }\int_{0}^{\omega_{\text{co}}}G_{\delta \delta }\left(\omega \right)d\omega }{\frac{1}{2\pi }\int_{0}^{\infty }G_{\delta \delta }\left(\omega \right)d\omega }=0.5$$

### 2.3 A proof of limitations in these methods

**Fig. 1:**
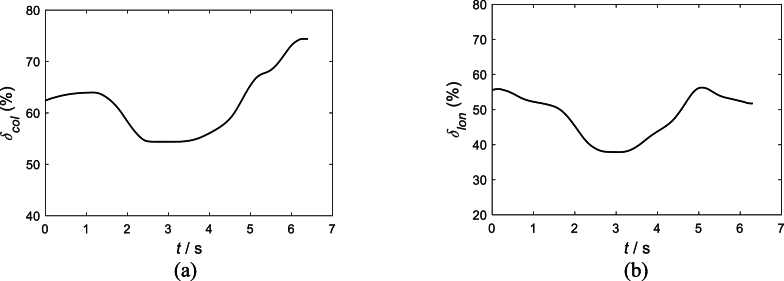
Pilot control inputs from UH-60A helicopter flight test data [16]: (a) Pilot collective stick input (%), (b) Pilot longitudinal cyclic stick input (%).

To demonstrate the limitations of current methods for assessing pilot control workload, we use these indicators to calculate three types of UH-60A helicopter maneuvers and compare them with the actual HQRs provided by the pilots. This section evaluates pilot workload using pilot control inputs from UH-60A helicopter flight test data [16] during landing procedures with one engine inoperative (OEI). The helicopter had a total weight of 7103 kg and was operating in standard atmospheric conditions. After completing the flight mission, the pilot assigned a Handling Quality Rating (HQR) of 4–5, indicating a Level 2 workload. During this flight test, the helicopter exhibited a significant single-engine failure landing maneuver, and the pilot's control frequency range was also wide. Therefore, the pilot's HQR value and subjective description can be used for comparative analysis. This paper examines the control inputs of the collective stick and the longitudinal cyclic stick, which indicate more aggressive pilot control actions, as shown in Fig. 1, where Fig. 1(a) represents the pilot collective stick input (%), and Fig. 1(b) represents the pilot longitudinal cyclic stick input (%).

Table 1 shows the aggressiveness and cut-off frequencies of pilot controls calculated based on the flight test data presented in Fig. 1. The data sampling frequencies are referenced from Ref. [[17], [18], [19]].

**Table 1:** Aggressiveness and cut-off frequencies of pilot controls calculated from UH-60A helicopter flight test data.

Method	$\delta_{col}\left(t\right)$	$\delta_{lon}\left(t\right)$
Aggressiveness	5.60 %	7.46 %
Cut-off frequency	1.0 (rad/s)	0.81(rad/s)

To illustrate the limitations of the two assessment methods of pilot control workload, we compare the aggressiveness of UH-60A helicopter ship landing operations and the pilot cut-off frequencies associated with UH-60A helicopter maneuvers (i.e., Bob-up/Bob-down and Dash/quick-stop) with the results presented in Table 1.

The ship landing maneuvers functioned as simulated instances for UH-60 helicopter missions involving approaches and touchdowns on FFG-7 Perry-class frigates [17]. The sea state alternated between calm conditions and sea state 4, which was characterized by the ship's motion due to sea waves in the absence of the ship's own velocity. The aircraft's task initiation involved a trimmed forward flight at 20 knots, with the starting point 1000 ft behind the ship and at a height of 100 ft above sea level. Pilots were instructed to make their approach towards the ship's stern at a closure rate between 10 and 20 knots, carry out a flare to achieve a stable hover for a duration of 8–12 s, and ultimately, accomplish a precise landing within a specific 5–10 feet square zone at the heart of the ship's deck.

Three pilots executed the maneuvers known as Bob-up/Bob-down and Dash/quick-stop, as outlined in the ADS-33E [18], utilizing UH-60 helicopters. These expert pilots then determined the corresponding pilot cut-off frequencies for these maneuvers [19]. Table 2 provides a comprehensive overview of the requirements specific to these maneuvers.

**Table 2:** Requirements for the maneuvers of Bob-up/Bob-down and Dash/quick-stop [19].

Maneuvers	Description
Bob-up/Bob-down	Starting from a stable hover at 40 ft above ground level, the vehicle swiftly ascends to 80 ft and achieves stability within 10 s. It then remains at 80 ft for 5 s before commencing its descent. The vehicle descends rapidly back to 40 ft above ground level and achieves stability within the subsequent 10 s. After achieving this stability, the vehicle maintains its hover position for an additional 20 s.
Dash/quick-stop	Initiating a 20 ft hover, rapidly accelerating to 60 knots, and promptly decelerating to achieve a quick-stop at a 20 ft hover within 30 s or less. Maintaining the stabilized hover for 20 s.

Table 3 provides a comparative analysis of the aggressiveness levels in two UH-60A helicopter ship landing operations and the pilot cut-off frequencies for two separate UH-60A helicopter maneuvers.

**Table 3:** The aggressiveness and the pilot cut-off frequencies of UH-60A helicopter maneuvers.

Method	Flight task	$\delta_{col}\left(t\right)$	$\delta_{lon}\left(t\right)$
Aggressiveness	Shipboard landing Calm sea state	7.5%–12 % [17]	4.0%–6.6 % [17]
	Shipboard landing Sea state 4	6.4%–14.5 % [17]	4.1%–8.1 % [17]
Cut-off frequency	Bob-up/bob-down	0.88–1.29(rad/s) [19]	0
	Dash/quick-stop	0	0.51–0.54(rad/s) [19]

Table 1, Table 3 demonstrate that the overall aggressiveness of pilot control actions during a UH-60A helicopter's OEI landing is nearly equivalent to the peak overall aggressiveness seen in UH-60 helicopter shipboard landings under sea state 4 conditions. The corresponding HQR for these shipboard landings falls within HQR 3 to 4 [17]. Furthermore, the pilot cut-off frequencies for both the UH-60A helicopter's OEI landing and the Bob-up/Bob-down maneuvers correspond to the moderate control processes that are characteristic of transport aircraft maneuvers, situating within the frequency band of 0.8–2.0 rad/s as mentioned in Ref. [3]. The Bob-up/Bob-down maneuver of the UH-60 helicopter is rated with an HQR between 2 and 3 [19].

Therefore, it is evident that the pilot workload indicators of aggressiveness and pilot cut-off frequency yield inconsistent HQR results. The former suggests that the UH-60A helicopter's pilot workload during OEI flight tests may fall within the Cooper-Harper HQR 3–4 range, while the latter indicates an HQR in the range of 2∼3. In fact, the actual HQRs obtained from pilots performing UH-60A helicopter OEI landing tasks indicate a higher workload (HQR 4–5) compared to other tasks. This comparison highlights the limitations of these methods.

In fact, it can be observed from equations (1), (2), (3), (4) that the pilot aggressiveness metric is obtained through time-domain analysis, which poses challenges in accurately reflecting the pilot control frequency. Additionally, the power spectral density and cut-off frequency of the pilot are derived from Fourier transformation, allowing for accurate identification of frequency components within the signal but unable to determine their specific timing. These traditional methods individually assess the pilot's control actions by considering the magnitude and frequency of the control inputs. However, these metrics represent normalized outcomes over time and do not directly capture the evolving complexity of pilot control.

## 3 Wavelet analysis-based evaluation method modeling

In response to the shortcomings of traditional metrics like pilot aggressiveness and cut-off frequency, which do not account for the temporal variations in control maneuvers, recent developments have brought forth novel approaches employing time-frequency domain representation. Prominent among these are the short-time Fourier transform (STFT) and the more modern wavelet analysis. While STFT encounters challenges in resolving frequency and time simultaneously, wavelet analysis—renowned for its superior time-frequency resolution—has increasingly gained favor in the engineering field [3,14]. This study primarily focuses on employing wavelet analysis to investigate pilot control actions, with the objective of evaluating and analyzing pilot control workload using this advanced analytical technique.

In contrast to the PSD, the time-frequency domain representation offers a comprehensive view of how signal energy is distributed across both the frequency and time dimensions. The wavelet analysis method uses finite-length bandpass filters, defined by a duration of $n_{c}/\omega_{c}$ seconds, with $n_{c}$ signifying the count of cycles. In the output of wavelet analysis, each data point reflects the weighted power $G_{\delta \delta }\left(\omega ,t\right)$ of the input signal $G_{x}\left(t\right)$ at a specific frequency $\omega_{c}$ within a time window of length $n_{c}/\omega_{c}$. This weighting is applied by the wavelet function, as further explained in Ref. [14].

(5) $$\int_{‐\infty }^{\infty }\psi \left(t\right)\text{dt}=0$$

Equation (5) represents the expression of the wavelet function. Selecting the appropriate wavelet function is crucial in wavelet transform due to the significant differences in waveform among the various functions available. As a result, this section explores the attributes of wavelet functions and their particular uses to identify the optimal choices. When it comes to signal recognition tasks, the selection criteria for wavelet functions include Support Set, Orthogonality, Regularity, and Vanishing Moments [15].

Taking into account these features, the wavelet function set named Daubechies, pioneered by Ingrid Daubechies, fulfills the criteria for analyzing pilot manipulation actions [15,21]. These functions are distinguished by their orthogonality, compact support, high regularity, and appropriate vanishing moments. As a result, this study selects Daubechies (db) wavelet functions for examining the pilot control actions.

Once a suitable wavelet function is chosen, the pilot's control input—representing a signal that varies instantaneously—is submitted to wavelet transformation. The wavelet family can be adjusted by a scaling factor (*s*) to vary the frequency and by a translation parameter (*u*) to shift in time. As a result, the wavelet function *ψ*(*t*) and its Fourier transform can be expressed as Equation (6),

(6) $$\left\{ \begin{array}{c} \psi_{u,s}\left(t\right)=\frac{1}{\sqrt{s}}\psi \left(\frac{t‐u}{s}\right)\\ {\hat{\psi }}_{u,s}\left(f\right)=e^{‐i2\pi \text{fu}}\sqrt{s\hat{\psi }}\left(\text{sf}\right) \end{array}\right.$$

The expression for the wavelet transform of the time-varying signal is as follows,

(7) $$W_{y}\left(u,s\right)=\int_{‐\infty }^{\infty }y\left(t\right)\psi_{u,s}^{*}\left(t\right)\text{dt}=\int_{‐\infty }^{\infty }\hat{y}\left(f\right)\psi_{u,s}^{*}\left(f\right)\text{df}$$

where $W_{y}\left(u,s\right)$ represents the wavelet coefficient, and ∗ denotes the conjugate relationship.

The wavelet transform, as defined by Equation (7), maximizes coefficients when the wavelet's center frequency aligns with specific components of the original signal. This characteristic gives the wavelet function properties similar to those of a bandpass filter, enabling it to selectively pass signals with frequencies near the wavelet's central frequency. Throughout the transformation, a spectrum of center frequencies is derived through scaling, while the translation coefficients capture the signal's varying time-frequency attributes. This dual mechanism reveals the signal's frequency and amplitude profiles at discrete time intervals. Hence, wavelet analysis is effective at pinpointing the primary frequency components within the pilot's control inputs.

According to the descriptions in Refs. [14,15,21], the Daubechies 3 (db3) wavelet is well-suited for analyzing pilot maneuvers due to its characteristics. It has finite support, which allows it to effectively capture local features in the signal. Its multi-resolution capability provides a detailed analysis at various scales, revealing both fine and broad aspects of the maneuver. Additionally, the smoothness and orthogonality of db3 aid in noise reduction, enhancing the clarity of the extracted features. Its computational efficiency makes it suitable for large-scale data analysis, which is crucial for monitoring and assessing pilot actions. Therefore, this paper selects the "db3″ function.

As described in Ref. [6], the maximum manipulation frequency of a pilot generally does not exceed 10.0 rad/s. This paper builds on this foundation by opting for a sampling rate of 20 Hz, as a higher sampling frequency does not enhance the accuracy of the wavelet transform but instead increases computational load.

Following this, the various predominant frequency components within pilot control inputs are detected by employing the approach suggested in Refs. [14,15].

1. To ascertain the frequencies associated with each instant during the maneuver, we pinpoint the peaks within the TFR at every moment in time. For example, Fig. 2(a) displays a vertical cross-section of the two-dimensional spectrogram at the 49-s mark, highlighting six significant peaks. It's crucial to mention that only local maxima in the spectrogram's magnitude that exceed a specific threshold are considered as peaks. To date, no precise theoretical standard has been put forward for selecting the specific threshold. In our research, we establish it at 8 % of the highest value in the continuous-time TFR, as reported in the work of references [14,15].

**Fig. 2:**
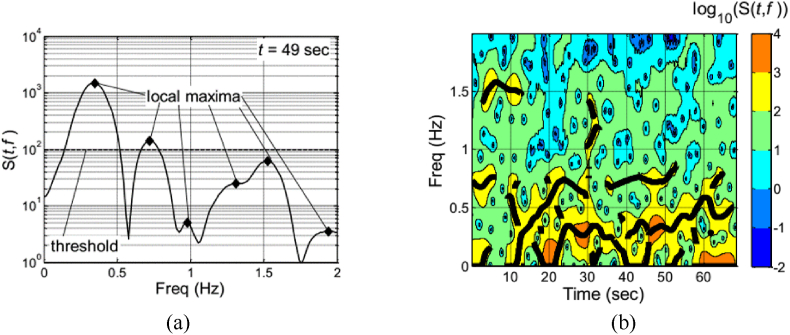
"Peaks" and "ridgelines" in the TFR of a Hover Mission Task, pilot lateral cyclic control input [15]: (a) "Peaks" at 49 s in the TFR, (b) "Ridgelines" in the TFR.
2. The next phase in the multi-frequency strategy entails the mathematical detection of successive local maxima over time, indicating sustained features within the TFR. Our focus is on identifying pairs of local maxima that occur at (or approximately at) the identical frequencies. In our research, we apply a threshold of 0.02 Hz to assess the continuity between two local maxima at successive time points. This procedure is designed to mathematically determine the "peaks" and "ridgelines" in the TFR's topographical depiction, as exemplified in Fig. 2(b).

It is worth noting that there is currently no specific mathematical derivation process for these two parameters, and references [14,15] also chose them based on actual simulation experience. However, for the first parameter, we found that the impact on the analysis results is not significant when the threshold is between 8 % and 30 %. For the second parameter, as long as it can reflect the continuous change of the "Ridgelines", the threshold can be chosen between 0.01 and 0.05 Hz without affecting the analysis results.

According to the work in Refs. [15,20,21], predominant frequency components typically align with different flight maneuvers or strategic controls, as detailed in Table 4. These frequency ranges are obtained by analyzing the control input signals of pilots during different flight missions. Through spectral analysis of the signals, researchers can identify frequency components associated with specific control strategies. For example, low-frequency components may be related to the pilot's trimming actions to maintain aircraft attitude, while high-frequency components may be related to control strategies requiring rapid response. It should be highlighted that these frequency bands are suggested based on the stick operations of fixed-wing aircraft pilots and might not be immediately relevant to other aircraft types or control inputs, such as the pedal controls in helicopters. However, it can be reasonably speculated that similar mappings between frequency control activities and control strategies may also exist in other types of aircraft. These frequency ranges provide a framework for analyzing pilot performance in flight tests and help explain their workload during specific tasks.

**Table 4:** Proposed frequency ranges for control tasks [15,20,21].

**Frequency Range**	Pilot Control Strategy/Task
0.25–0.8 rad/s (0.04–0.13 Hz)	Typical open-loop control associated with trimming and flight path modulation
0.8–2.0 rad/s (0.13–0.32 Hz)	Typical closed-loop control associated with transport aircraft maneuvering
2.0–4.0 rad/s (0.32–0.64 Hz)	Higher-gain closed-loop control associated with increased task urgency or handling issues with the aircraft, such as Pilot Induced Oscillations (PIO)
4.0–10.0 rad/s (0.64–1.59Hz)	Very high-gain closed-loop control, almost certainly associated with control difficulties

**Fig. 3:**
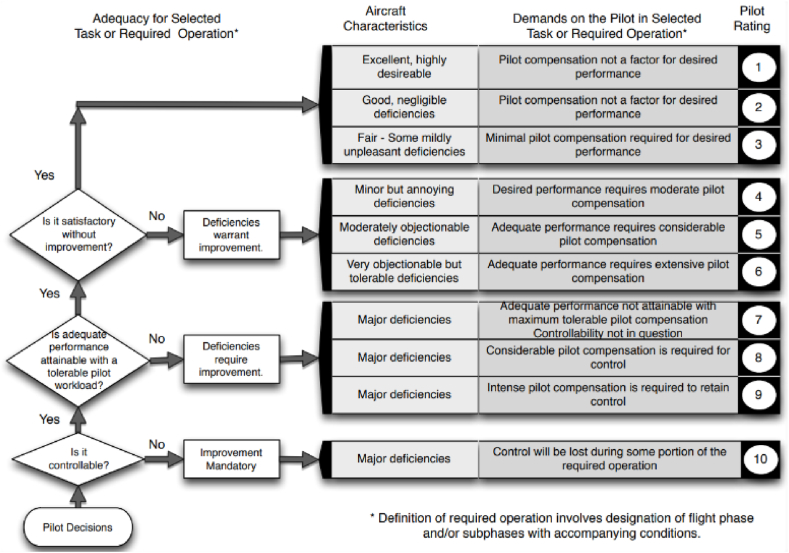
Cooper-Harper Handling Qualities Rating Scale [21].

Upon examination, one can discern a definite association between the control strategy outlined in Table 4 and the pilots' HQR ratings on the Cooper-Harper scale, as depicted in Fig. 3 [21].

Upon scrutinizing the pilot control strategies presented in Table 4 alongside the HQR levels depicted in Fig. 3, a possible link emerges between the frequency bands and the HQR levels. In particular, the frequency band from 0.25 to 0.8 rad/s is preliminarily linked to HQR Level 1, corresponding to scores of 1–3. The band from 0.8 to 2.0 rad/s is related to HQR Level 2, with scores ranging from 4 to 6. The 2.0–4.0 rad/s band is connected to HQR Level 3, encompassing scores of 7–9. Lastly, the 4.0–10.0 rad/s band is associated with an HQR rating of 10, which signifies the most challenging control tasks, as detailed in Table 5.

**Table 5:** Potential mapping relationship between Table 4 and the Cooper-Harper HQR Scale.

**Dominant Frequency Range**	Pilot Control Strategy/Task	HQR Scale
0.25–0.8 rad/s	Typical open-loop control associated with trimming and flight path modulation	Level 1 (1–3)
(0.04–0.13Hz)		
0.8–2.0 rad/s	Typical closed-loop control associated with transport aircraft maneuvering	Level 2 (4–6)
(0.13–0.32 Hz)		
2.0–4.0 rad/s	Higher-gain closed-loop control associated with increased task urgency or handling issues with the aircraft, such as PIO	Level 3 (7–9)
(0.32–0.64 Hz)		
4.0–10.0 rad/s	Very high-gain closed-loop control, almost certainly associated with control difficulties	10
(0.64–1.59 Hz)		

Thus, the assessment approach for pilot control workload is delineated as such: To begin with, the wavelet transform is utilized to distill the primary frequency components from the pilot's control maneuvers. Subsequently, the pilot's workload level and potential HQR range are predicted by correlating these frequency components with the descriptions of the control strategies and the Cooper-Harper scale. This method offers a comprehensive description of pilot inputs in terms of amplitude and frequency, and subsequently quantifies these inputs using the HQR, thereby merging qualitative and quantitative analytical perspectives. In the context of research on pilot control strategies for manned eVTOL aircraft, it enhances the understanding of pilot control behavior.

## 4 Case study

### 4.1 Case study for helicopter landing in OEI

Given the absence of publicly available pilot control data and corresponding workload evaluations for eVTOL aircraft, this study uses a flight test of the UH-60A helicopter's single-engine failure safe landing as a case study [16]. The determination of the level of pilot's control workload and the possible HQR range relies on the pilot control inputs documented throughout the landing phase. This evaluation is then compared with the pilot's actual HQR to validate the rationale behind the proposed pilot control workload assessment technique.

According to the flight test details [16], the initial altitude for the OEI landing is 13.7 m, the forward velocity is 5.1 m/s, the heading is set at 0°, and the sideslip angle is also at 0°. The helicopter's total weight is 7103 kg, and it operates in a standard atmospheric environment. After completing the flight mission, the pilot assigned the HQR a rating of 4–5. In the flight test, the helicopter exhibited significant maneuver amplitudes during the single-engine failure landing, and the pilot's control frequencies varied widely. The pilots also provided HQRs and subjective descriptions of their control inputs, making the data suitable for comparative analysis.

In this study, we analyze the control inputs for the collective lever, ranging from 0 to 100 percent, and the longitudinal cyclic lever over the same range, which signifies more forceful maneuvers. Following the sampling process, the initial stick deflection, which occurs without any control input, is corrected by subtracting the initial displacement. This adjustment sets the energy value of the signal to zero at the onset of the analysis, ensuring an accurate baseline. In conducting the wavelet transform, the "db3″ wavelet function is utilized, given its established effectiveness in a wide array of applications. We primarily focus on analyzing frequency components that significantly exceed the threshold (e.g., surpassing 30 % of the maximum value), thereby minimizing the impact of high-frequency, low-energy density values that may represent measurement noise.

**Fig. 4:**
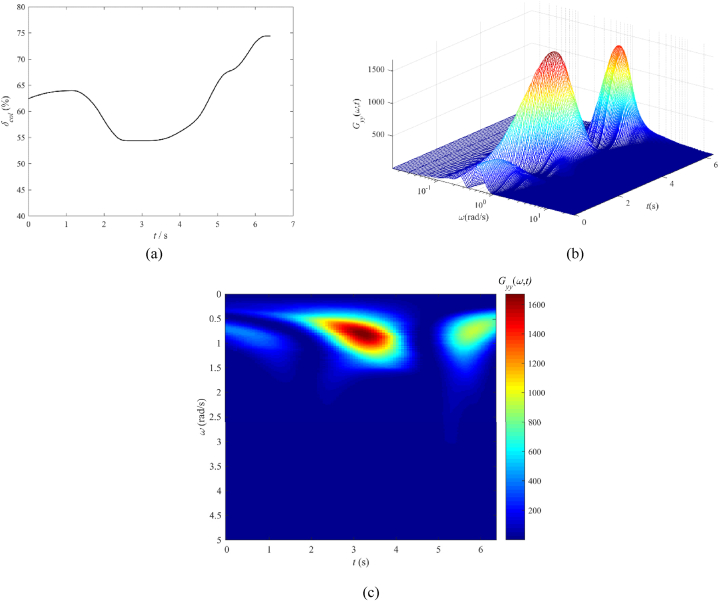
Collective control input and wavelet transform result, UH-60A helicopter OEI landing: (a) Pilot collective control input (%), (b) Three-dimensional plot of the wavelet transform result, and (c) Two-dimensional top-down view of the wavelet transform result.

Fig. 4 illustrates the time-based sequence of the collective control input in conjunction with the results from the wavelet transform. Fig. 4(a) represents the pilot collective stick input (%). In Fig. 4(b), ω denotes the frequency of control input in radians per second (rad/s), and $G_{yy}\left(\omega ,t\right)$ represents the signal energy distribution across both the frequency spectrum and the time domain, quantified in units of %^2^/(rad/s). Fig. 4(b) shows the three-dimensional outcome of the wavelet transform, which is then transformed into a two-dimensional top-down view as seen in Fig. 4(c), facilitating the identification of energy input and frequency components. From Fig. 4(c), it can be observed that there are two noticeable instances of significant pilot manipulation. The first manipulation exhibits a frequency range concentrated between 0.5 and 1.3 rad/s with a large magnitude, while the second manipulation shows a frequency range concentrated between 0.4 and 1.2 rad/s with a smaller magnitude. These two manipulations reflect the pilot's operations of lowering the collective lever for descent and rotational speed maintenance, followed by the action of increasing the collective lever to slow down and achieve a landing. Comparing this with Tables 5 and it can be inferred that the collective lever manipulation aligns with the maneuvering characteristics of transport aircraft. Additionally, at 2.5 s and 5.2 s, there are two high-frequency components exceeding 2 rad/s, but with extremely low energy intensity (indicating minimal pilot manipulation or measurement noise). This suggests that the current flight task has a relatively low level of urgency.

We also observe that the cut-off frequency of the pilot's collective stick, as calculated using equation (4), falls precisely between the two primary frequency components of the pilot (*ω*_*co*_ = 1.0 rad/s). This is because the previously described cut-off frequency of the pilot represents a synthesis of energy and frequency components obtained through wavelet analysis. Therefore, in comparison to the conventional cut-off frequency of the pilot, results derived from wavelet analysis offer additional insights into manipulation frequencies and energy.

**Fig. 5:**
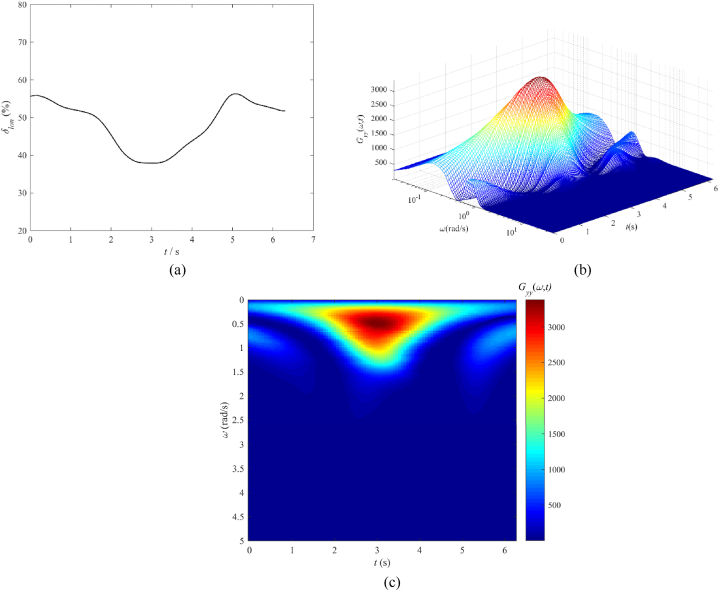
Longitudinal cyclic control input and wavelet transform result, UH-60A helicopter OEI landing: (a) Pilot longitudinal cyclic control input, (b) Three-dimensional plot of the wavelet transform result, and (c) Two-dimensional top-down view of the wavelet transform result.

Fig. 5 presents the timeline and wavelet transform outcome of the longitudinal stick input provided by the pilot, where Fig. 5(a) represents the pilot longitudinal stick input (%), and Fig. 5(b) represents the three-dimensional plot of the wavelet transform result. Notably, the longitudinal stick input demonstrates higher maximum energy values relative to the collective stick, suggesting a more extensive manipulation range.

From the perspective of frequency components in Fig. 5(c), the pilot demonstrates a significant frequency band ranging from 0.2 to 1.4 rad/s, which happens roughly between the 2–4 s. Compared with Table 5, frequencies below 0.8 rad/s are linked to balance and flight path adjustments, whereas the band from 0.8 to 2.0 rad/s pertains to maneuvers typical of transport aircraft. This suggests that the pilot adjusts the flight path while controlling the helicopter's nose-down acceleration and nose-up deceleration during landing using the longitudinal stick. At 5–6 s, there exists a low-energy frequency component corresponding to the pilot's adjustments in flight attitude when touching down. In addition, two high-frequency components are detected at 2.5 and 5.2 s, exceeding 2 rad/s but with very low energy intensity (indicating tiny manipulation by the pilot or measurement noise). This also suggests that the flight mission has relatively low urgency. Similarly, Equation (4) indicates that the pilot's cut-off frequency for the longitudinal stick is 0.81 rad/s, and this is within the spectrum of frequencies that the wavelet transform includes.

In conclusion, the analysis of various frequency components suggests that the OEI landing operation is predominantly characteristic of maneuvers typical of conventional transport aircraft, which are associated with a relatively lower degree of flight urgency. According to the HQR rating description in Fig. 3 and Table 5, the pilot's workload may fall closer to the medium-high assessment value of level 2. This aligns with the ratings provided by pilots during actual flights (HQR 4–5, at level 2), which suggests that the method for assessing pilot control workload developed in this research is justified.

### 4.2 Case study for tilt-rotor aircraft conversion

In this part, we employ the forward conversion simulation of the XV-15 tilt-rotor aircraft, as referenced in Ref. [15], to confirm the effectiveness of our suggested approach. The conversion simulation is based on an optimal control model. The optimized trajectory and control strategy simplify the pilot's task by allowing small adjustments to the collective stick for rotor force modulation, the longitudinal stick for attitude adjustment, and fine-tuning for system trimming. As shown in Table 5, the pilot's manipulation during the XV-15 conversion primarily involves trimming control with appropriate maneuvering. This paper further examines the pilot's manipulation using wavelet transform to predict workload level and potential HQR range based on the correlation between control amplitude, frequency components, and defined frequency ranges for control tasks.

**Fig. 6:**
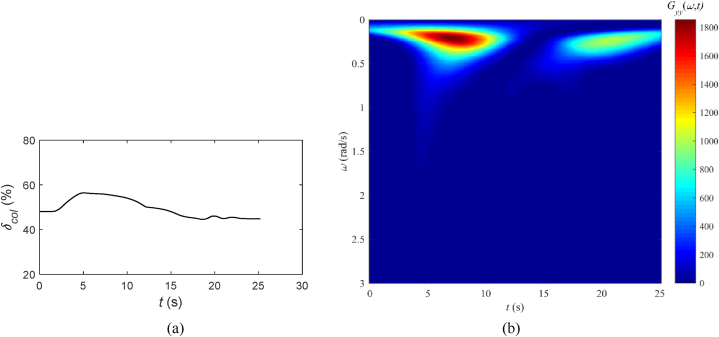
Collective control input and wavelet transform result, tilt-rotor aircraft conversion simulation: (a) Pilot collective control input, (b) Two-dimensional top-down view of the wavelet transform result.

Fig. 6 presents the time-history and wavelet transform result of pilot collective control input, where Fig. 6(a) represents the pilot collective control input (%), and Fig. 6(b) represents the two-dimensional top-down view of the wavelet transform result.

Fig. 6(b) depicts a significant high-energy input within the frequency band from 0.1 rad/s to 0.6 rad/s, which takes place between 2 s and 10 s. Following this, at approximately 17 s, there is a slight adjustment in amplitude near 0.3 rad/s, which is associated with the fine-tuning of the collective pitch stick. Moreover, it is observed that the high-frequency components with low energy, indicated by small amplitudes, are all below 1.7 rad/s, signifying minimal levels of energy and amplitude.

**Fig. 7:**
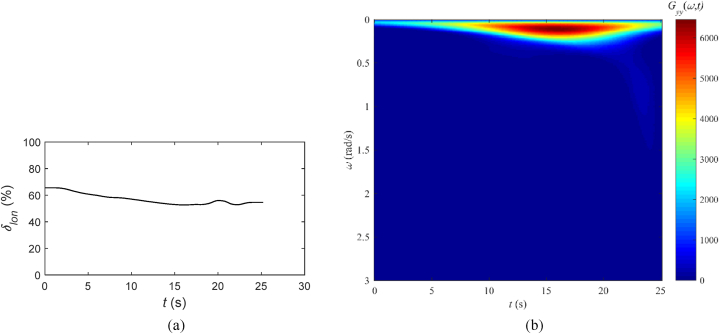
Longitudinal control input and wavelet transform result, tilt-rotor aircraft conversion simulation: (a) Pilot longitudinal control input (%), (b) Two-dimensional top-down view of the wavelet transform result.

Fig. 7 shows the time-history and the result of wavelet transform of the pilot longitudinal control input, where Fig. 7(a) represents the pilot longitudinal control input (%), and Fig. 7(b) represents the two-dimensional top-down view of the wavelet transform result.

Fig. 7(b) demonstrates that the main energy input from the longitudinal stick is focused on frequencies under 0.3 rad/s, aligning with the adjustment of the aircraft's flight attitude. Following this, after 22 s, a secondary high-frequency component with low energy emerges, with frequencies not exceeding 1.6 rad/s, signifying the start of the final trim control phase.

Referring to Table 5 from Section 3, one can deduce that the pilot's manipulation includes trim and flight path adjustments, predominantly involving energy inputs linked to frequency components that are below 0.8 rad/s. A minor fraction of the energy inputs is attributed to maneuvering actions typical of transport aircraft, occurring within the frequency range of 0.8–2.0 rad/s. According to the correlation between frequency components and workload outlined in this paper, it is probable that the pilot's control workload is situated somewhere within the range of Level 1 and Level 2 on the HQR scale, corresponding to a rating of 3–4. This assessment suggests that the forward conversion process is associated with a low workload and is considered relatively straightforward for the pilot, which closely matches the results in Ref. [22] regarding the assessment of pilot workload during the conversion phase of tilt-rotor aircraft.

## 5 Conclusions

This study presents an innovative evaluation method for assessing the workload of pilots during eVTOL aircraft operations. Despite the continued reliance on the Cooper-Harper rating (HQR, Handling Quality Rating) as a benchmark for workload assessment, the subjective nature of the HQR has limited the precision and quantifiability of workload analysis. To tackle this problem, we propose a wavelet transform-based method that measures pilot workload by analyzing the relationship between control magnitude, frequency components, and the intricacy of control actions. It correlates pilot control actions, as characterized by wavelet transform, with the Cooper-Harper Control Quality Rating (HQR) scale, providing a more objective basis for evaluating pilot workload in eVTOL aircraft operations.

Our method is validated through case studies involving a flight test of UH-60A helicopter single-engine failure safe landing and a simulation of tilt-rotor aircraft conversion. The results demonstrate that this method accurately identifies the frequency components and energy levels of pilot control actions across various time intervals, and the provided pilot workload indicator (HQR) aligns with pilots' reports. Therefore, the developed approach offers a rational framework for quantifying and analyzing pilot workload in a variety of eVTOL aircraft scenarios.

In conclusion, our study contributes to the field of eVTOL aircraft operations by providing a more objective and analytical method for evaluating pilot workload. Subsequent studies should concentrate on broadening the use of this method to include actual eVTOL flight data and to enhance the link between workload assessment and control strategies, with the objective of improving pilot efficiency and safety.

## Funding

This research is supported by the 10.13039/501100001809National Natural Science Foundation of China (No. 12202406).

## Data availability statement

Research-related Data is not stored in publicly available repositories, and Data will be made available on request.

## Ethics declarations

Review and/or approval by an ethics committee is not needed for this study because we don't work with humans or animals.

## CRediT authorship contribution statement

**Lijuan Hu:** Writing – original draft. **Xufei Yan:** Writing – review & editing, Supervision, Resources, Conceptualization. **Ye Yuan:** Writing – review & editing, Investigation.

## Declaration of competing interest

The authors declare that they have no known competing financial interests or personal relationships that could have appeared to influence the work reported in this paper.

## References

[1] Çinar E., Tuncal A. (2023). A Comprehensive analysis of society's perspective on urban air mobility. Journal of Aviation.

[2] Pavel M.D. (2022). Understanding the control characteristics of electric vertical take-off and landing (eVTOL) aircraft for urban air mobility. Aero. Sci. Technol..

[3] Mello R., Klyde D., Mitchell D. (23–27 January 2023). Proceedings of the AIAA SCITECH 2023 Forum, AIAA.

[4] Shepherd M., Macdonald A., Gray W. (Nov 2009). IEEE Aerospace Conference, Big Sky.

[5] Hanson C., Schaefer J., Burken J. (2014).

[6] Ji H., Chen R., Li P. (2017). Rotor-state feedback control to alleviate pilot workload for helicopter shipboard operations. J. Guid. Control Dynam..

[7] Memon W., White M., Padfield G. (2022). Helicopter Handling Qualities: a study in pilot control compensation. Aeronaut. J..

[8] Fujizawa B., Lusardi J., Tischler M. (May 3–5, 2011). Response type tradeoffs in helicopter handling qualities for the GVE. American Helicopter Society International 67th Annual Forum Proceedings, Virginia Beach, VA: AHS.

[9] Gray W. (Aug 2005). AIAA Atmospheric Flight Mechanics Conference and Exhibit Proceedings.

[10] Jones J., Padfield G., Charlton M. (1999). Wavelet analysis of pilot workload in helicopter low level flying tasks. Aeronaut. J..

[11] Lampton A., Klyde D. (2012). Power frequency: a metric for analyzing pilot-in-the-loop flying tasks. J. Guid. Control Dynam..

[12] Klyde D., Schulze P., Thompson P. (May 2010).

[13] Tritschler J., O'Connor J. (2016). Use of time-frequency representations for interpreting handling qualities flight test data. J. Guid. Control Dynam..

[14] Tritschler J., O'Connor J., Klyde D. (May 2017). Proceedings of the 73rd Annual Forum of American Helicopter Society.

[15] Yan X., Yuan Y., Chen R. (2023). Research on pilot control strategy and workload for tilt-rotor aircraft conversion procedure. Aerospace.

[16] Nagata I., Mittag C., Skinner G. (1976). Government Competitive Test, Utility Tactical Transport Aircraft System (UTTAS), Sikorsky YUH-60A Helicopter. Final Report.

[17] Soneson G., Horn J., Zheng A. (2016). Simulation testing of advanced response types for ship-based rotorcraft. J. Am. Helicopter Soc..

[18] Alabama. Handling qualities requirements for military rotorcraft (2000). United States Army Aviation and Missile Command Aviation Engineering Directorate Redstone Arsenal No.: ADS-33E-PRF.

[19] Atencio J. (1993).

[20] Field E., Giese S. (Aug 2013). AIAA Atmospheric Flight Mechanics Conference & Exhibit.

[21] Klyde D.H., Pitoniak S.P., Schulze P.C. (2020). Piloted simulation evaluation of tracking mission task elements for the assessment of high-speed handling qualities. J. Am. Helicopter Soc..

[22] Yu X., Chen R., Wang L. (2022). An optimization for alleviating pilot workload during tilt rotor aircraft conversion and reconversion maneuvers. Aero. Sci. Technol..

